# Investigating the effects of beta-amyloid on hippocampal signalling in Alzheimer's disease

**DOI:** 10.1186/1471-2202-16-S1-P34

**Published:** 2015-12-18

**Authors:** Julia M Warburton, Daniel J Whitcomb, Krasimira Tsaneva-Atanasova, Kei Cho

**Affiliations:** 1 Bristol Centre for Complexity Sciences, University of Bristol, Bristol, BS8 1TR, UK; 2 Henry Wellcome Laboratories for Integrative Neuroscience and Endocrinology (HW-LINE), University of Bristol, Bristol, BS1 3NY, UK; 3 Centre for Synaptic Plasticity, University of Bristol, Bristol, BS1 3NY, UK; 4 Department of Mathematics, University of Exeter, Exeter, EX4 4QF, UK

## 

Alzheimer's disease (AD) is the leading form of dementia and is characterised clinically by cognitive decline and impairments to memory function. The protein amyloid-β (Aβ) is thought to be a key mediator of this neurodegeneration [1]. Mounting evidence suggests that soluble Aβ_1-42 _oligomers (oAβ_1-42_) cause the impairments observed in early AD, which includes synapse loss and synaptic dysfunction. For example, oAβ_1-42 _inhibits hippocampal long-term potentiation (LTP) [2], an important synaptic plasticity thought to underlie memory formation in the brain [3]. We have recently found that postsynaptic infusion of oAβ into hippocampal neurons causes an increase in synaptic conductance attributed to changes in the excitatory glutamate receptor profile at the synapse. The relevance of this to synaptic dysregulation and consequence on hippocampal signalling is yet to be determined.

Here we take a computational approach to investigate how oAβ_1-42 _dysregulates signalling within the hippocampus. We first develop a biophysical model of synaptic potentiation in a CA1 neuron, based on a simple kinetic synapse model and Hodgkin-Huxley formalism [4], which successfully reproduces the conductance increase observed in single-cell patch clamp experiments following oAβ application. We then extend this to investigate how this affects signalling across a small network of CA1 neurons, analysing the importance of connectivity strength and number of affected neurons to the overall dynamics of the system. Recent studies have suggested that there are two distinct types of CA1 neurons with different electrophysiological properties [5]. Therefore we also consider the impact a heterogeneous population would have on signalling across the network.

## References

[B1] Hardy J Selkoe DJ The amyloid hypothesis of Alzheimer's disease: progress and problems on the road to therapeutics Science 2002 297 5580 353 356 12130773 10.1126/science.1072994

[B2] Walsh D Klyubin I Fadeeva JV Cullen WK Anwyl R Wolfe MS Rowan MJ Selkoe DJ Naturally secreted oligomers of amyloid β protein potently inhibit hippocampal long-term potentiation in vivo Nature 2002 416 6880 535 539 11932745 10.1038/416535a

[B3] Bliss TV Collingridge GL A synaptic model of memory: long-term potentiation in the hippocampus Nature 1993 361 6407 31 39 8421494 10.1038/361031a0

[B4] Destexhe A Mainen ZF Sejnowski TJ An efficient method for computing synaptic conductances based on a kinetic model of receptor binding Neural Comput 1994 6 1 14 18

[B5] Mizuseki K Diba K Pastalkova E Buzáski G Hippocampal CA1 pyramidal cells form functionally distinct sublayers Nature Neurosci 2011 14 1174 1181 21822270 10.1038/nn.2894 PMC3164922

